# Septal Preparation for TAVR Using Radiofrequency Catheters in Patients With LVOT Obstruction: 1-Year Follow-Up

**DOI:** 10.1016/j.jaccas.2025.103606

**Published:** 2025-06-11

**Authors:** Bruno P. Valdigem, Antonio Tito Paladino Filho, Andrea de Andrade Vilela, Edileide de Barros Correia, Auristela Ramos, Jorge Eduardo Assef, Dimytri Siqueira, Ibraim Masciarelli Pinto Filho

**Affiliations:** Instituto Dante Pazzanese de Cardiologia, Sao Paulo, Brazil

**Keywords:** septal ablation, septal preparation, septal reduction therapy, suicide ventricle

## Abstract

Left ventricular outflow tract (LVOT) obstruction is frequent in hypertrophic cardiomyopathy. Septal radiofrequency ablation for treatment of LVOT obstruction, which is endocardial radiofrequency ablation of septal hypertrophy (ERASH), was previously performed in patients refractory to medical treatment with catheters used for cardiac arrhythmia ablation. Obstruction of the LVOT during transcatheter aortic valve replacement (TAVR) procedures may lead to suicide ventricle, the dire event of a sudden increase in the intraventricular gradient after valve obstruction alleviation. We present a 2-step procedure for septal preparation before TAVR in a series of 4 cases. All patients underwent ERASH before TAVR, which successfully reduced the LVOT gradient before implant (the longest waiting interval between procedures was 3 months and the shortest was 15 days). No patient underwent pacemaker or implantable cardioverter-defibrillator implant. To the best of our knowledge, this is the longest series of septal ablation before TAVR in patients who also presented with intraventricular LVOT obstruction.

## Graphical Abstract

**Figure:**
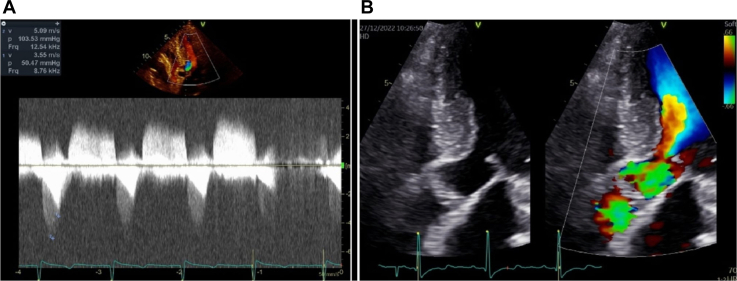

Left ventricular outflow tract (LVOT) obstruction is frequent in hypertrophic cardiomyopathy and can lead to heart failure, angina, and syncope.^1^ Endocardial radiofrequency ablation of septal hypertrophy (ERASH) for treatment of LVOT obstruction was previously performed in patients refractory to medical treatment using a transthoracic needle approach^2^ and therapeutic catheters used for treatment of ventricular arrhythmias.^3^^,^^4^ It has also been used to increase LVOT size, allowing mitral valve transcatheter intervention.^5^ Obstruction of the LVOT may present as a challenge for patients with aortic valve stenosis who are candidates for transcatheter aortic valve replacement (TAVR) procedures because of the dire event of a sudden increase in the intraventricular gradient after valve obstruction alleviation.^6^ That event is known as suicide ventricle. We present a 2-step procedure using catheter radiofrequency septal preparation before TAVR implant as a solution to prevent suicide ventricle in a series of cases.

**Take-Home Messages**

• Left ventricle obstruction may pose an additional threat in patients undergoing TAVR and should be addressed before valve intervention.
• Endocardial radiofrequency ablation may be used as a means to prepare the patient by removing LVOT obstruction regardless of coronary anatomy and site of obstruction (in contrast to alcohol septal ablation).

## Methods

Patients were selected as candidates for TAVR who presented with intraventricular obstruction; gradients above 30 mm Hg either in rest or Valsalva maneuver; symptoms of heart failure, angina, or syncope; and refractory to available medications (beta-blockers and diuretics). Because it was not possible to assess the contribution of each component of the obstruction, we considered the gradient as a marker for obstruction severity and as a risk factor for suicide ventricle, deeming prior treatment.

**Figure 1:**
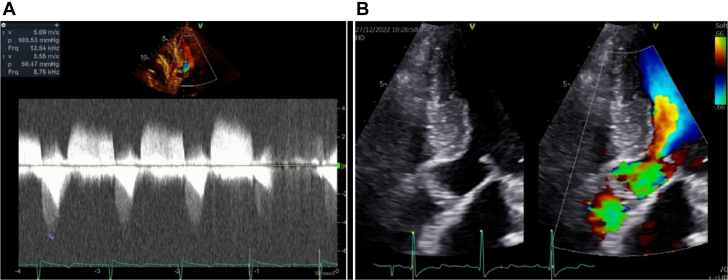
From Patient 4, Before Interventions (A) Two distinct curves are highlighted: curve 2 in dark gray is related to aortic valve stenosis and the smaller curve 1 in brighter gray is related to the left ventricular outflow tract gradient. (B) Aliasing (flow acceleration) begins inside the left ventricle, and the systolic anterior movement of the mitral valve can also be seen.

Gradients that arise from LVOT obstruction and aortic valve obstruction may not be easily distinguished. In most cases, we manage to find 2 distinct curves using spectral Doppler, as seen in Figure 1A. In cases in which both curves were not clear, the presence of flow acceleration inside the left ventricle with systolic anterior movement of the mitral valve and characteristic mitral reflow (Figure 1B) were diagnostic of LVOT obstruction.

**Figure 2:**
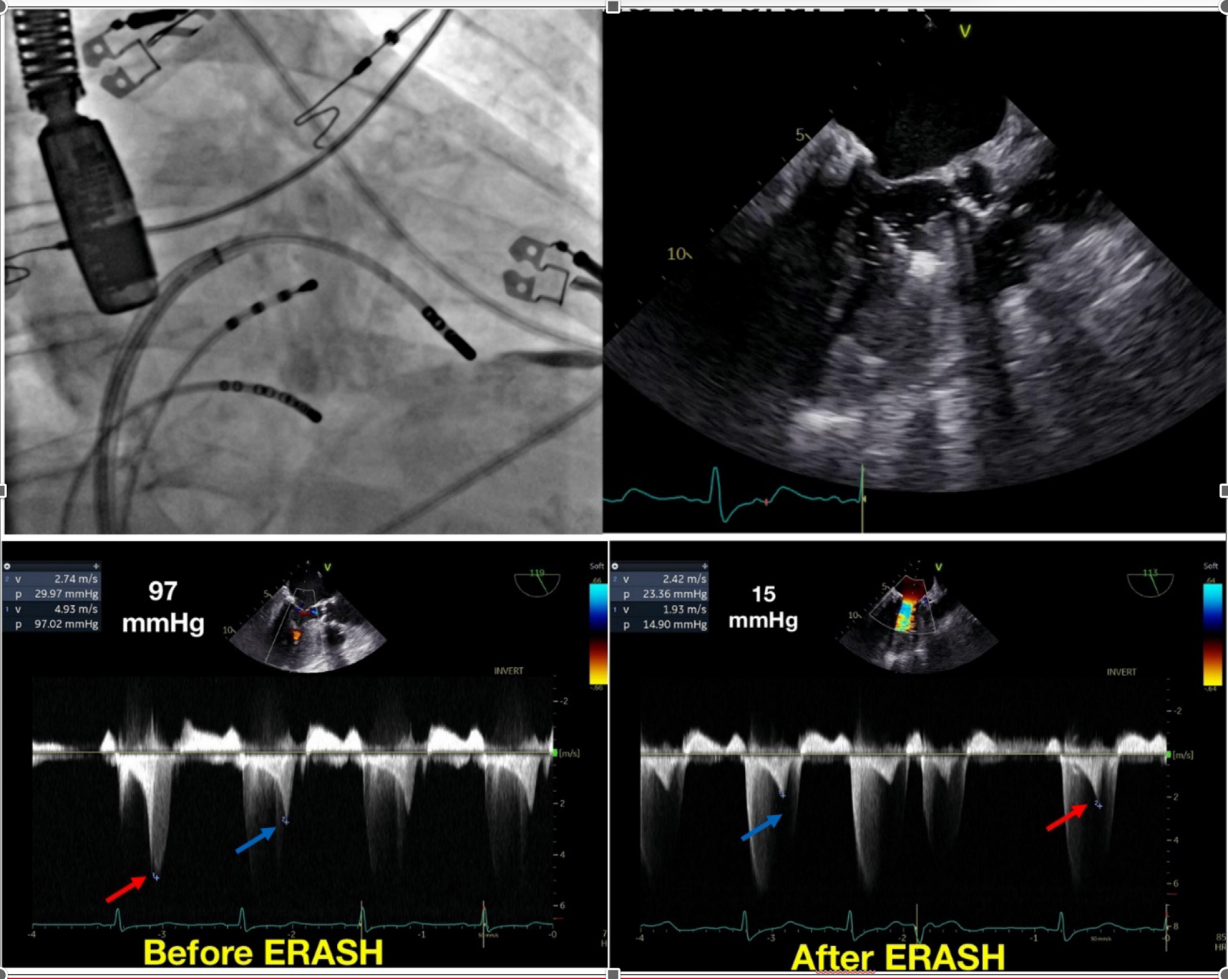
ERASH for Septal Preparation Before Transcatheter Aortic Valve Replacement (Top left) Fluoroscopic view of the ablation catheter. (Top right) Transesophageal echocardiogram view of catheter ablation of the left ventricular septum. (Bottom left) Maximum left ventricular outflow tract (LVOT) gradient after the ventricular premature beat (red arrow) and basal conditions (blue arrow) before ERASH. (Bottom right) Maximum LVOT gradient after the ventricular premature beat (red arrow) and basal conditions (blue arrow) after ERASH. ERASH = endocardial radiofrequency ablation of septal hypertrophy.

All patients had a computed tomography of the heart and transthoracic echocardiogram to confirm the level of obstruction and anatomy feasibility for the procedures. Ablation was performed with the patient under general anesthesia guided by transesophageal echocardiogram, and the target was the left ventricular septum in the vicinity of the aliasing. Because of aortic valve disease, all ablations were performed using transseptal puncture and deflectable sheets (Figure 2).

Gradients were measured once more using transesophageal echocardiogram after general anesthesia and in 2 cases using a ventricular premature beat (VPB) to enhance the maximal gradient (provoked gradient). Radiofrequency was delivered using 8-mm catheters with a power of 60 to 80 W and impedance carefully controlled to avoid steam pops. A His bundle catheter was placed in the tricuspid valve to increase the safety of the procedure and another in the coronary sinus to identify more promptly junctional rhythms of atrioventricular dissociation. All patients were in the intensive care unit for 48 hours and the hospital for another 48 hours (as per protocol in our institution). TAVR procedures were performed at discretion of the interventional cardiologist.

The procedures were performed by the same operators and according to protocol were registered in the Brazilian registry of clinical trials (Plataforma Brasil CAAE: 72754617.0.0000.5462). All patients signed informed consent after careful explanation of risks and benefits.

## Case 1

Our first patient was a 74-year-old woman with a mean aortic valve gradient of 51 mm Hg, aortic valve area of 0.34 cm^2^/m^2^, peak velocity of 4.63 cm/s, and an LVOT maximum gradient of 86 mm Hg after Valsalva maneuver. The ERASH procedure was performed using the gradient obtained after a VPB as the maximum LVOT gradient (97 mm Hg), and by the end of the procedure, the gradient after VPB was reduced to 15 mm Hg. Three month after ERASH, the patient underwent a successful transcatheter aortic valve implantation procedure, without any residual intraventricular LVOT gradient. The final transaortic gradient was 5 mm Hg and aortic valve area 1.8 cm^2^. After 1 year, the patient remained asymptomatic and without an intraventricular gradient (Figure 2). Video 1 shows complete case 1 details.

## Case 2

Our second patient, a 73-year-old woman, underwent balloon valvuloplasty on June 14, 2022 that was interrupted by a sudden increase in the intraventricular gradient to 108 mm Hg and hemodynamic instability (probable of suicide ventricle). The patient was referred to ERASH on June 23, 2022, with an LVOT gradient reduction during the procedure from 83 to 13 mm Hg. The patient already had a left bundle branch block, and the QRS increased from 156 ms to 183 ms and the His bundle ventricular interval changed from 40 ms to 36 ms. TAVR with a Sapien 3 #20 (Edwards) was implanted on October 6, 2022 successfully (additional safety measures for coronary protection were used in this case because of a low right coronary ostium implant. The intraoperative intraventricular gradient measured 5 mm Hg (28 mm Hg after VPB). At the last follow-up on April 8, 2024, the patient was asymptomatic with a moderate aortic leak. The aortic valve prosthesis gradient was 33 mm Hg (maximum) and 16 mm Hg (mean). There was no intraventricular gradient and a septum thickness of 12 mm.

## Case 3

Our third patient was an 87-year-old man with an aortic valve gradient of 81 mm Hg (maximum) and 50 mm Hg (medium) and an aortic valve area of 0.6 cm^2^. The LVOT resting gradient was 32 mm Hg with a septal thickness of 14 mm. The patient underwent ERASH on June 9, 2022 with an LVOT gradient reduction during the procedure from 45 mm Hg to 22 mm Hg (post-VPB gradient from 75 mm Hg to 38 mm Hg). One incidental left bundle branch block occurred, and the QRS increased from 114 ms to 145 ms and the His bundle ventricular interval from 48 ms to 40 ms. The patient underwent TAVR implant on July 7, 2022 (Sapien 3 #23) successfully. At the 1-year follow-up (August 17, 2023), the patient was asymptomatic, with an aortic valve area gradient of 21 mm Hg (maximum) and 12 mm Hg (median). No LVOT gradient was found, and the septum thickness was 12 mm.

## Case 4

Our fourth patient, a 69-year-old woman, was being tested for a suspected abdominal neoplastic disease (recurrent ascites). She also had aortic stenosis (maximum gradient: 83 mm Hg; mean: 76 mm Hg; aortic valve area: 0.9 cm^2^) and an intraventricular LVOT maximum gradient of 51 mm Hg. She underwent ERASH on January 10, 2023, with an LVOT gradient reduction from 36 mm Hg to 18 mm Hg during the procedure. Because of the urgency of a possible treatable comorbidity, we performed the TAVR implant on January 26, 2023 with a Myval n24.5 (Meril Life Sciences Pvt. Ltd) successfully. The intraventricular gradient was 17 mm Hg during the procedure. At a routine follow-up on April 8, 2024, the patient presented with atrial fibrillation that spontaneously returned to sinus rhythm on the next day. A follow-up echocardiogram was performed 1 week after and did not show an LVOT gradient; the 1-year follow-up also did not show an LVOT gradient.

**Figure 3:**
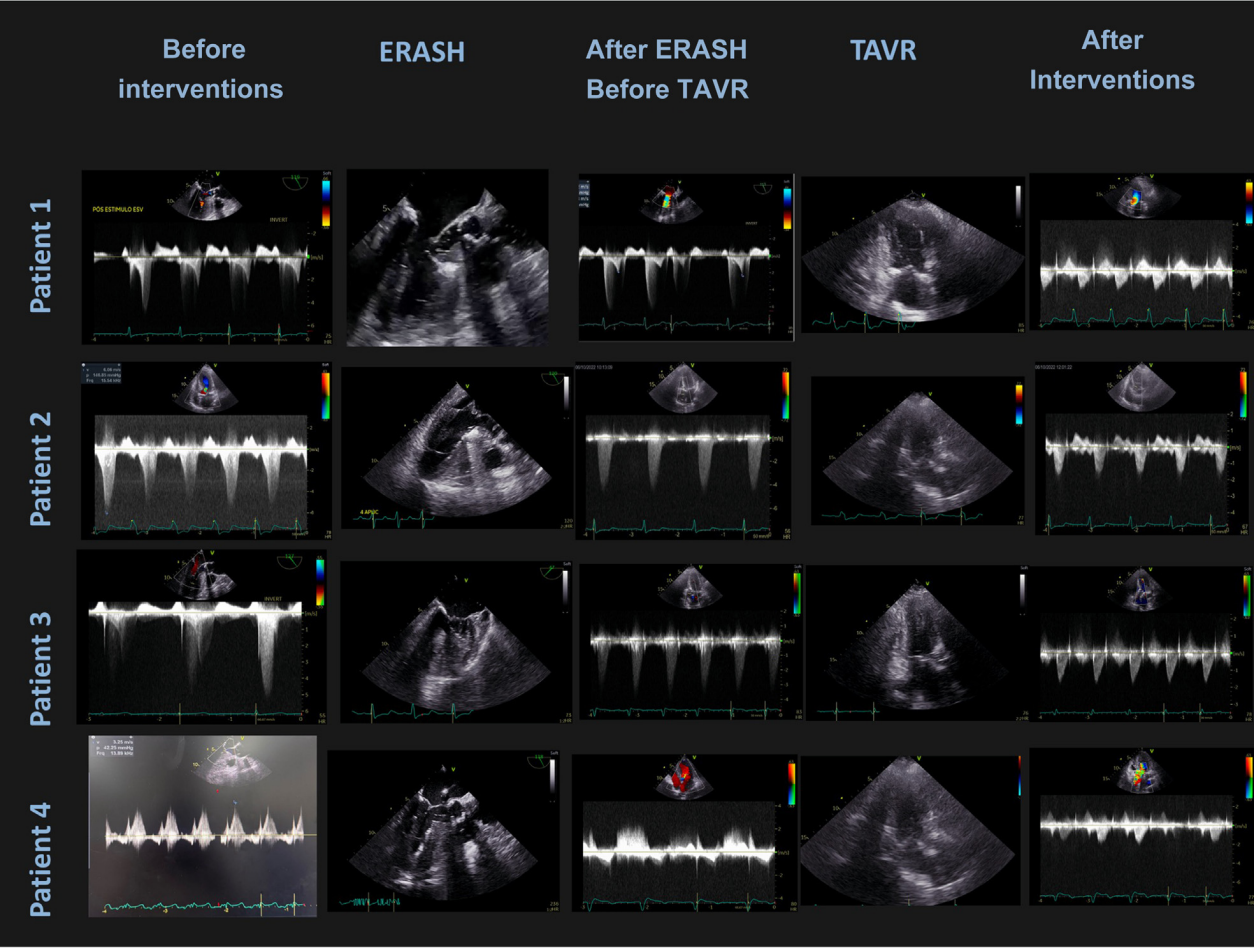
Complete Echocardiographic Images of the 4 Cases in Each Step of Treatment Images were acquired for each patient in the different steps of the treatment. The images in the first column present echocardiography before interventions, with 2 distinct peaks of obstruction: one earlier representing valve obstruction and one later “dagger-shaped” representing LVOT obstruction. The second column shows TEE images taken during septal ablation. It is possible to visualize the catheter inside the LVOT. The third column shows the absence of LVOT gradient before TAVR, but significant aortic valve gradient remains. The fourth columns shows an intraoperatory TAVR TTE image. The last column shows the gradients after all interventions were successfully performed. LVOT = left ventricular outflow tract; TAVR = transcatheter aortic valve replacement; TEE = transesophageal echocardiogram; TTE = transthoracic echocardiography.

No patient underwent pacemaker implant or had significant complications (stroke, myocardial infarction, or bleeding) during the first year of follow-up. For complete echocardiographic images of the 4 cases, see Figure 3. The patients characteristics and follow up are presented in Tables 1 and 2.

**Table 1:** Patient Characteristics

	Patient 1	Patient 2	Patient 3	Patient 4
Demographics				
Sex	F	F	M	F
Age, y	78	73	89	72
Body surface area, m^2^	1.39	1.51	1.84	1.41
Clinical symptoms				
Chest pain or tightness	Y	Y	Y	Y
Shortness of breath	Y	Y	Y	Y
Syncope or presyncope	N	Y	N	N
NYHA functional classification				
II		Y		
III	Y		Y	Y
IV				
Family history of hypertrophic cardiomyopathy	N	N	Y	Y
Hypertension	Y	Y	Y	Y
Coronary artery disease	Y	N	Y	N
Atrial fibrillation	N	N		N
Diabetes	Y	N	Y	N
Cerebrovascular accident	N	Y	N	N
Medications				
Beta-blocker	N	N	Y	Y

**Table 2:** Procedure Characteristics

	Patient 1	Patient 2	Patient 3	Patient 4
Septal hypertrophy without other segments	Y	Y	Y	Y
Ablation				
Ablation procedure time, min	180	150	120	125
Radiofrequency delivery duration, min	18	14	15	20
Maximum power, W	70	70-80	60-80	60-80
LVOT gradient rest, mm Hg	29	83	45	36
LVOT gradient provoked, mm Hg	74	NA	72	NA
LVOT gradient final, mm Hg	0	13	22	18
LVOT gradient provoked final, mm Hg	0	NA	38	NA
Septum	17	15	15	16
48-h gradient, mm Hg	9	11	30	10
Transcatheter aortic valve replacement				
Time between procedures, wk	12	12	4	2
TAVI aortic stenosis mean gradient, mm Hg	100	45	45	67
TAVI aortic stenosis mean final gradient, mm Hg	6	5	4	7
LVOT gradient, mm Hg	0	23	0	17
Type of prosthesis	Sapien 3 #20	Sapien 3 #20	Sapien 3 #23	Myval#24,5
Septum	13	13	12	16
1-y follow-up				
Mean atrioventricular gradient	5	16	12	4
LVOT gradient	0	0	0	0

LVOT = left ventricular outflow tract; NA = not available; TAVI = transcatheter aortic valve implantation.

## Discussion

Suicide left ventricle is a life-threatening adverse event that may occur when the aortic valve stenosis gradient is suddenly reduced, leading to paradoxical hemodynamic collapse due to an increase in the intraventricular gradient.^7^ The clinical features are a hemodynamic collapse or severe hypotension related to a paradoxical increase in the intraventricular gradient immediately after TAVR deployment. A systematic review of 25 articles on acute hemodynamic compromise after TAVR (also known as suicide ventricle) reported that patients were usually women with septal hypertrophy, small ventricles, and hyperdynamic contractility in the assessment immediately before the TAVR procedure.^8^ Emergency ethanol septal ablation is frequently used as a bail-out alternative,^9^ but identification and treatment of precipitating factors, such as treatment of septal hypertrophy and obstruction (“septal preparation”), before TAVR might avoid the hemodynamic compromise.

ERASH is a recent technique to reduce the LVOT gradient through radiofrequency delivery to septal portions of the LVOT using therapeutic electrophysiology catheters.^3^^,^^4^^,^^10^ It was recently recommended in Brazilian guidelines for treatment of hypertrophic cardiomyopathy as an alternative for patients who were not candidates for ethanol ablation or surgical myectomy.^11^ ERASH has also been used in children in a small study^12^ with good results, because ethanol ablation is usually not recommended in the pediatric population and surgical myectomy in children requires expertise.

ERASH was also used in patients with small neo-LVOT obstructions to allow the transcatheter treatment of the mitral valve.^5^ ERASH was used as a means to reduce septal thickness and increase LVOT volume. In our study, there was a reduction in septal thickness, but the small number of patients might not provide us with a definite answer on the significance of that finding. In a recent review and meta-analysis, the findings of most studies was consistent with a reduction in the septal thickness to around 4 mm.^13^ That result should be carefully interpreted because 2 methods of radiofrequency ablation were used, intramyocardial in two-thirds of the patients and endocardial in the rest. Significant septal reduction was mostly observed in intramyocardial ablations (through direct chest puncture of the heart using a radiofrequency needle, creating great myocardial destruction).^14^ In our previous publication^3^ and in this case series, it was noteworthy that the movement of the septum toward the LVOT was different in our 4 patients after ablation. The previous “buldging” of the septum changed, and the area of fibrosis or hypokinesis of the ablated region would move like a wall, pulled by the surrounding structures. Most patients in the main series presented edema of the LVOT that persisted on transthoracic echocardiogram for several weeks. We also noted that the reduction of the LVOT gradient could be delayed until the third month, and in rare cases the gradient would continue to improve until the sixth month. We chose to wait for 3 months in the first patient, but because of symptoms and improvement of the echocardiographic parameters by the end of first month we believe a shorter interval between procedures is feasible if the echocardiographic parameters are acceptable.

We did not perform computed tomography after the ablation and before TAVR. It was not described in the research protocol for ERASH in our institution or in TAVR protocols. Also, patient 3 had chronic kidney disease (serum creatinine: 1.7 mg/dL). Patient 4 needed to undergo an extensive evaluation for possible neoplastic syndrome and thus would be exposed to other radiation-based procedures. It was decided by investigators that echocardiographic parameters should be used for follow-up and further clinical decision.

Echocardiography-guided ERASH for treatment of LVOT obstruction is part of an ongoing single-center research at our institution. All patients with LVOT obstruction provide informed consent and are selected to their treatment of choice after consultation with the heart team for the best strategy of LVOT obstruction relief. Direct myosin inhibitors are not available in yet in Brazil. The main advantage over ethanol septal ablation is that favorable coronary anatomy is not relevant to this procedure or to the site of obstruction. Damage to the conduction system is prevented by mapping and recognition of the His bundle and left bundle branch. It is possible to evaluate the need for a pacemaker during the same procedure, because the expansion of the lesion and late atrioventricular block are not expected with ERASH.

To the best of our knowledge, this is the longest follow-up of ERASH before TAVR in patients who also present with intraventricular LVOT gradient. Because suicide ventricle is a lethal complication of TAVR procedures, radiofrequency ablation may be present as an easy way to reproduce the technique. We believe training echocardiographers in a wider array of septal reduction techniques may provide interventional cardiologists with resources to treat more patients with minimally invasive procedures.

## Conclusions

Septal preparation of patients with aortic stenosis and LVOT intraventricular obstruction using radiofrequency catheters is feasible and may provide a new strategy to prevent suicide ventricle in TAVR candidates.

## Funding Support and Author Disclosures

The authors have reported that they have no relationships relevant to the contents of this article to disclose.

## Appendix

Video 1 Step by Step Approach to Patient Selection, Radiofrequency Ablation and Transcatheter Aortic Valve Replacement Procedure
